# CNS GNPDA2 Does Not Control Appetite, but Regulates Glucose Homeostasis

**DOI:** 10.3389/fnut.2021.787470

**Published:** 2021-11-29

**Authors:** Ruth Gutierrez-Aguilar, Bernadette E. Grayson, Dong-Hoon Kim, Suma Yalamanchili, Mario L. Calcagno, Stephen C. Woods, Randy J. Seeley

**Affiliations:** ^1^División de Investigación, Facultad de Medicina, Universidad Nacional Autónoma de México (UNAM), Mexico City, Mexico; ^2^Laboratorio de Enfermedades Metabólicas: Obesidad y Diabetes, Hospital Infantil de México “Federico Gómez”, Mexico City, Mexico; ^3^Department of Neurobiology and Anatomical Science, University of Mississippi Medical Center, Jackson, MS, United States; ^4^Department of Pharmacology, Korea University College of Medicine, Seoul, South Korea; ^5^College of Medicine, University of Cincinnati, Cincinnati, OH, United States; ^6^Laboratorio de Fisicoquímica e Ingeniería de Proteínas, Departamento de Bioquímica, Facultad de Medicina, Universidad Nacional Autónoma de México (UNAM), Mexico City, Mexico; ^7^Department of Psychiatry and Behavioral Neuroscience, University of Cincinnati, Cincinnati, OH, United States; ^8^North Campus Research Complex, Department of Surgery, University of Michigan, Ann Arbor, MI, United States

**Keywords:** glucosamine-6-phosphate deaminase 2, glutamine-fructose-6-phosphate aminotransferase (GFAT), glucose homeostasis, third ventricle of the hypothalamus, appetite control

## Abstract

*GNPDA2* has been associated with human obesity and type-2 diabetes by using a GWAS approach. GNPDA2 is an enzyme involved in the hexosamine biosynthesis pathway, which is known to be important for nutrient sensing in various organism. Its counter enzyme, GFAT, has previously been shown to be important to the development of insulin resistance in diabetes. The implication of GNPDA2 and GFAT in metabolism is scarce and the effect of both enzymes over appetite and glucose homeostasis is unknown.

**Aim:** Identify the role of GNPDA2 and GFAT in nutrient sensing circuits of the CNS that are important for the regulation of both appetite and glucose homeostasis.

**Methods:** Using Long Evans rats, we administered either a GNPDA2 or GFAT antagonist or vehicle in *i3vt*.

**Key Findings:** *GNPDA2* is highly expressed in hypothalamus and adipose tissue, followed by muscle and liver. *GNPDA2* is expressed in different hypothalamic nuclei (ARC, DMH, LHA, PVN). *GNPDA2* is downregulated in hypothalamus under diet-induced obesity (as previously described), but *GFAT* expression does not change. Moreover, *i3vt* infusion of GNPDA2 or GFAT inhibitor resulted in increased c-Fos in areas related to appetite and glucose homeostasis control as PVN and DMH and to a lesser extent in the LHA and ARC. Central inhibition of GNPDA2 does not alter either acute food intake or body weight; however, GFAT inhibition diminished appetite and body weight due to visceral illness. In addition, central administration of the GNPDA2 antagonist, prior to an intraperitoneal glucose tolerance test, resulted in glucose intolerance in comparison to vehicle without altering insulin levels.

**Significance:** These results suggest that central GNPDA2 does not control appetite, but regulates glucose homeostasis.

## Introduction

A growing body of evidence points to an important role of the central nervous system (CNS), in particular the hypothalamus, to regulate energy balance and glucose homeostasis. The hypothalamus can sense and integrate peripheral signals such as hormones and nutrients and make responses to maintain both energy and glucose homeostasis (1).

The hexosamine biosynthesis pathway (HBP) is a key nutrient-sensing pathway (2). The first and rate-limiting enzyme of the HBP is glutamine-fructose-6-phosphate aminotransferase (GFAT); GFAT catalyzes the transamination between glutamine and fructose-6-phosphate (Fru6P) to form glucosamine-6-phosphate (GlcN6P) and glutamate. Under hyperglycemic conditions, GlcN6P is formed and rapidly converted to UDP-*N*-acetylglucosamine (UDPGlcNAc), which post-transcriptionally modifies various proteins through glycosylation (2). Acute central glucosamine infusion has also been demonstrated to augment the feeding response during low glucose levels (3). In addition, infusing glucosamine into the third ventricle of the hypothalamus (i3vt) produces glucose intolerance (4), demonstrating the importance of this pathway in glucose homeostasis. Moreover, the overexpression of GFAT in skeletal muscle and adipose tissue of mice results in insulin resistance (5, 6). Bypassing the GFAT step by administration of glucosamine provokes insulin resistance in 3T3-L1 cell line (7).

While much is known about the impact of GFAT in metabolism, little is known about glucosamine-6-phosphate deaminase 2 (GNPDA2), the enzyme operating on the opposite direction of GFAT. GNPDA2 is the enzyme that catalyzes the deamination of the GlcN6P to Fru6P and ammonia. Genome-wide association studies (GWAS) have revealed an association of GNPDA2 with human obesity, as well as with type-2 diabetes (8–10).

Recently, GNPDA2 has been implicated in adipogenesis and it alters the transcriptome profile of human adipose-derived mesenchymal stem cells, suggested to regulate lipid and glucose metabolism (11). However, its role in metabolism and its implication in obesity and type 2 diabetes is not well understood.

For this reason, our interest to understand the function of new genes associated with obesity and type 2 diabetes led us to analyze *GNPDA2* expression in the hypothalamus of diet-induced obese rats showing it is down-regulated (12). Therefore, this points to the possibility that CNS GNPDA2 has a role as part of the nutrient sensing pathways that regulates appetite and glucose homeostasis.

The current work aims to test the role of central GNPDA2 and GFAT on food intake and glucose homeostasis. We observed that central GFAT inhibition reduced food intake and body weight, but that may be potentially a secondary effect of visceral illness, since we also observed a conditioned taste aversion. We also found that inhibition of GNPDA2 does not alter food intake or body weight, but it does worsen glucose homeostasis.

## Materials and Methods

### Animals and Diets

**Figure 1:**
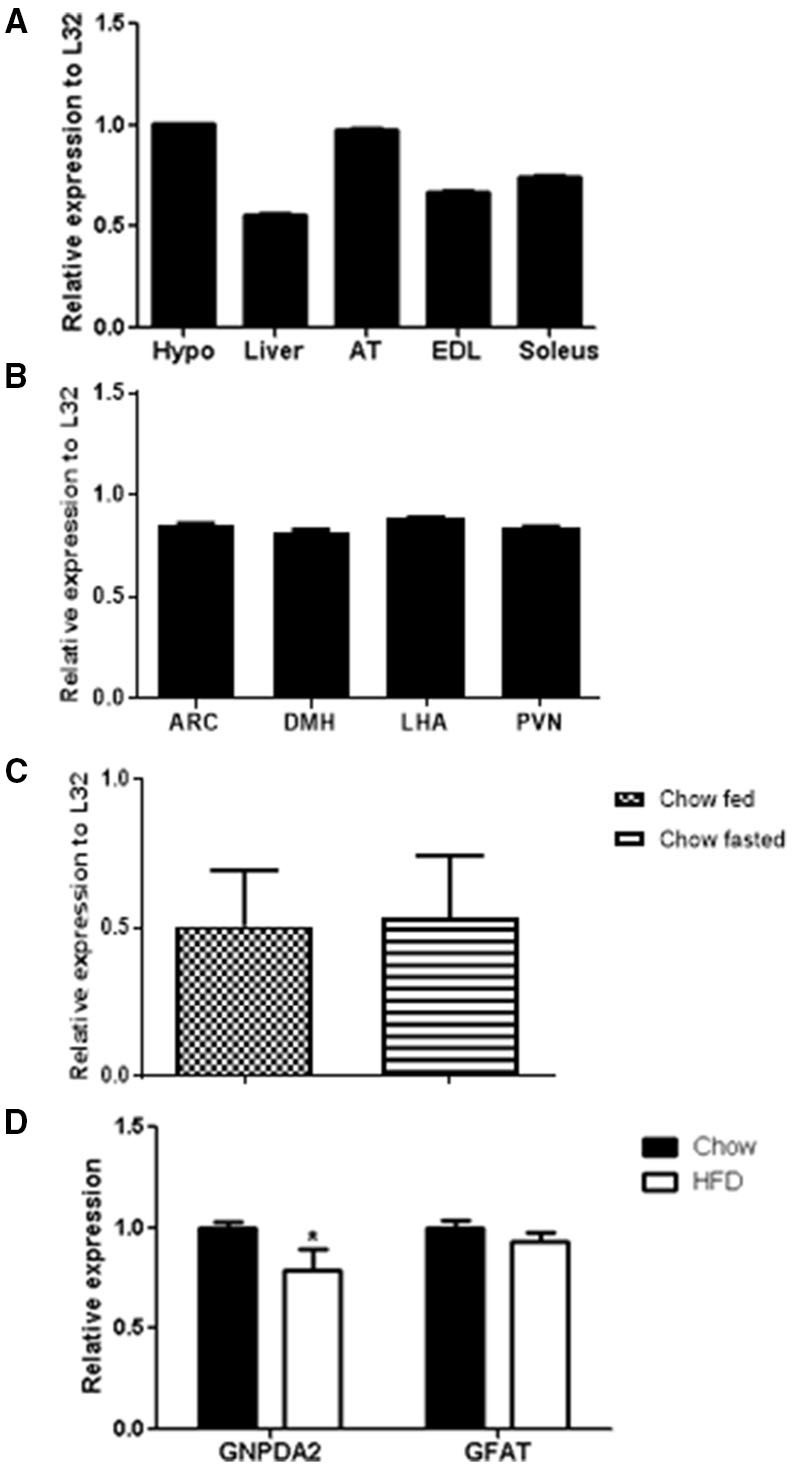
*GNPDA2* mRNA expression levels in different tissues and diets. **(A)** *GNPDA2* mRNA levels in hypothalamus (hypo), liver, adipose tissue (AT), extensor digitorum longus (EDL) and soleus muscle. **(B)** *GNPDA2* mRNA levels in arcuate nucleus of the hypothalamus (ARC), dorsomedial nuclei of the hypothalamus (DMH), lateral hypothalamic area (LHA) and paraventricular nuclei (PVN). **(C)** Hypothalamic *GNPDA2* mRNA levels of fasted (48 h) vs. fed (2 h) animals under chow diet. **(D)** Hypothalamic *GNPDA2* and *GFAT* mRNA levels on chow and high-fat diet. Relative expression calculated by the ΔΔCt method was normalized by *L32*, a housekeeping gene. The expression data were analyzed using a *t*-test ^*^P < 0.05. *n* = 15 rats per group.

Male Long-Evans rats (Harlan Labs, Indianapolis, IN, 250–350 g) were individually housed in tub cages and maintained on a 12/12-h light/dark cycle (lights on at 1,200 h). Rats were fed either pelleted chow (Harlan-Teklad, Indianapolis, IN) or a high-fat butter oil-based diet (HFD 40%, Research Diets D03082706, New Brunswick, NJ.) and allowed *ad libitum* access to water and their respective diets for 6 weeks for experiment showed in Figure 1. The rats were euthanized in fed (2 h) or fasted (48 h) conditions, as previously reported (12).

For the rest of the experiments, the animals were fed with chow diets. All procedures were approved by the University of Cincinnati Institutional Animal Care and Use Committee (IACUC).

### Hypothalamic Gene Expression

Micropunches of different areas of the hypothalamus were performed and RNA was extracted by a Qiagen miniprep (Qiagen, Valencia, CA). cDNA was synthetized by iScript cDNA synthesis kit (Bio-Rad Laboratories,Hercules, CA). RT-PCR was performed using SYBR green and *GNPDA2* primers (forward GCAGCCAAGTACATCTGCAA and reverse AGCTTTCGGGATGGTTTCTT), *GFAT* primers (forward GCCAGCGACTTCTTGGATAG and reverse CAAACATCACAAGGGACACG) and *L32* primers (forward CAGACGCACCATCGAAGTTA and reverse AGCCACAAAGGACGTGTTTC) as described elsewhere (12). The samples were run in triplicate. The mRNA levels were normalized to *L32* levels, a constitutively expressed ribosomal gene (housekeeping gene). The expression analysis was performed using the ΔΔCt method.

### Drugs

The GFAT inhibitor, DON (6-diazo-5-oxonorleucine), was obtained from Sigma-Aldrich and the GNPDA2 inhibitor (2-amino-2-deoxy-D-glucitol-6-phosphate or D-glucitolamine 6-phosphate, GlcNol6P) was synthesized as described by (13). Both drugs were diluted in saline.

### Surgical Procedures

Stainless steel cannulas were implanted in the 3rd-cerebral ventricle of the hypothalamus (i3vt) as described previously (14). Correct placement of each cannula was confirmed by i3vt injection of 10 ng angiotensin II (American Peptides, Sunnyvale, CA). A minimum of 5 mL of water consumption within 1 h of administration of angiotensin II was required for animals to remain in the study.

### Food Intake Experiments

Rats were fed with chow diet during these experiments. The HBP is a nutrient sensing pathway; therefore, we analyzed the impact of these enzymes on acute food intake. The hoppers were weighed 4 h prior to dark and were removed, in order to increase the sensitivity to detect appetite control without causing compensatory hyperphagia. In addition, the hoppers were removed to avoid any short bouts prior to the experiment and to let the animals to calm down until the injection.

To study the role of these enzymes over appetite control, an acute i3vt injection of the inhibitors was given 1 hour prior to dark. Saline (vehicle), a dose-response curve for GFAT inhibitor (75 and 150 μg) or GNPAD2 inhibitor (100 and 200 μg) was administered via Hamilton syringe via the i3vt cannula in 2.5 μL. Doses were chosen to be on the pharmacological window and based in different papers (15, 16). Based on the dose-response experiments, a dose of 150 μg was chosen for both drugs on further experiments. Hoppers were returned to the cage at the onset of the dark and food intake measured at 1, 2, 4, and 24 h. Body weight was measured before and 24 h after injection.

### Conditioned Taste Aversion Test (CTA)

To determine if the anorectic effect of the GFAT inhibitor was caused by visceral illness, a conditioned taste aversion test was performed, as previously described (17).

Rats were water deprived for 24 h and were given access for 2 h to a 0.15% saccharin solution (presented on the opposite side where the water bottle is normally placed). No water was available during that time. Saline, GFAT inhibitor (i3vt) or LiCl (0.15 M in a volume equivalent to 2% of each animal's body weight, i.p.) was injected, followed by removing the saccharin bottle and replacing the water bottle to its original location. The following day, animals were deprived of water for 24 h. Then, water and saccharin bottles were returned and placed on their respective sides of the cage. Fluid intake was measured at 2, 4, and 24 h. LiCl was used as a positive control for a CTA response.

### Glucose Tolerance Test (GTT)

Rats fed with chow diet were fasted for 16 h and infused i3vt with saline, GFAT inhibitor (150 μg) or GNPDA2 inhibitor (150 μg) 1 h prior to starting a GTT. Intraperitoneal glucose (1.5 mg/g of body weight) was injected and blood glucose levels were measured at 0, 15, 30, 45, 60, and 120 min after glucose administration in duplicate samples using Accu-chek glucometers and test strips (Roche, Indianapolis, IN). Blood was collected from the tail vein at indicated time points for measurement of plasma insulin (ELISA, Crystal Chem, Downers Grove, IL).

### c-Fos Immunohistochemistry

Rats fed with chow diet were perfused with 0.9% saline and 4% paraformaldehyde and the brains were removed and post-fixed overnight at 4°C. Hypothalamic sections (35 μm) were cut serially 1:6 by using a sliding-freezing microtome. Sections were stored at −20°C in cryoprotectant solution (30% sucrose in PBS) until time of use. Five washes in 0.1M PBS were performed followed by 9:1 methanol and 3% hydrogen peroxide for 15 min and rinsed in PBS. The sections were preincubated for 1 h at room temperature in blocking buffer (PBS plus 0.4% Triton X – 100 plus 2% normal donkey serum). Incubation with c-Fos specific antibody (1:2,500; Santa Cruz Biotechnology, Santa Cruz, CA; #sc-52) in blocking buffer for 48 h at 4°C was followed by several washes in PBS. Biotinylated donkey anti-rabbit antibody (1:300; Vector Laboratories, Burlingame, CA; # BA-1000) was incubated for 1 h. Washes and incubation with avidin-biotin solution (1:600; Vector ABC, VectorLaboratories; #PK-6100) were performed for 30 min. Sections were exposed to 3,3'-diaminobenzidine (DAB) enhanced with nickel chloride. Sections were mounted on gelatin coated slides, dried and coverslipped in Permount (Thermo Fisher, Waltham, MA, USA) after graded ethanol dehydration.

High-resolution images were taken by a blinded investigator using a Zeiss Axioplan 2 Microscope using a 4× objective and Zeiss Axiocam camera and Axiovision 4.8 Software. The arcuate nucleus (ARC), paraventricular nucleus (PVN), dorsomedial nucleus (DMH), and lateral hypothalamus area (LHA) were identified based on the Rat Brain Atlas by Paxinos. The focus was the following for each of the areas: ARC (Bregma −2.28 to −3.12), PVN (Bregma −1.08 to −2.08), DMH (Bregma −2.28 to −3.12), and LHA (Bregma −2.92 to −3.12). Three matched sections, right and left side (*n* = 6 sampling) of each brain region were obtained for each animal. The sampling area was identified using a standardized shape used as a region of interest and applied to each image. Scion Image version 4.0.3.2 was used to subtract background and analyze the number of objects (cFos positive cells) above background. The average number of positive cells per image is reported, as described (18). Representative images of each nuclei reported are provided at computerized 40× magnification. Scale bar = 100 μm.

### Statistical Analysis

All values are reported as mean ± SEM. Data were analyzed using ANOVA or repeated measures ANOVA, followed by Tukey's *post-hoc* tests or by *t*-Test as indicated. Analysis was performed using Prism (GraphPad Software, Inc., San Diego CA). Significance was set at *p* < 0.05, two-tailed, for all analyses.

## Results

### Relative Expression of *GNPDA2* in Different Tissues

*GNPDA2* has been reported to be highly expressed in human hypothalamus (9). We asked whether *GNPDA2* is expressed in different rat tissues fed with chow diet and if high-fat feeding alters its expression. *GNPDA2* was expressed in several tissues, including liver, extensor-digitorum longus (EDL) and soleus muscle. Interestingly, it was highly expressed in the hypothalamus and adipose tissue (Figure 1A).

To start understanding the role of central GNPDA2 in appetite and glucose homeostasis control, we also analyzed *GNPDA2* expression in different nuclei of the hypothalamus (ARC, PVN, DMH and LHA) that regulate both roles, and we found that the transcript is expressed similarly in all of them (Figure 1B).

As the HBP is known to sense nutrients, we then investigated if expression of *GNPDA2* could be modified by feeding/fasting conditions and HFD. We observed no difference under fasted or fed conditions on chow diet (Figure 1C). As we reported previously (12), *GNPDA2* is down-regulated in the hypothalamus of rats fed HFD in comparison to chow diet. Interestingly, *GFAT* expression was not different between these two conditions (Figure 1D).

### c-Fos Expression After Central Administration of GFAT and GNPDA2 Inhibitors

**Figure 2:**
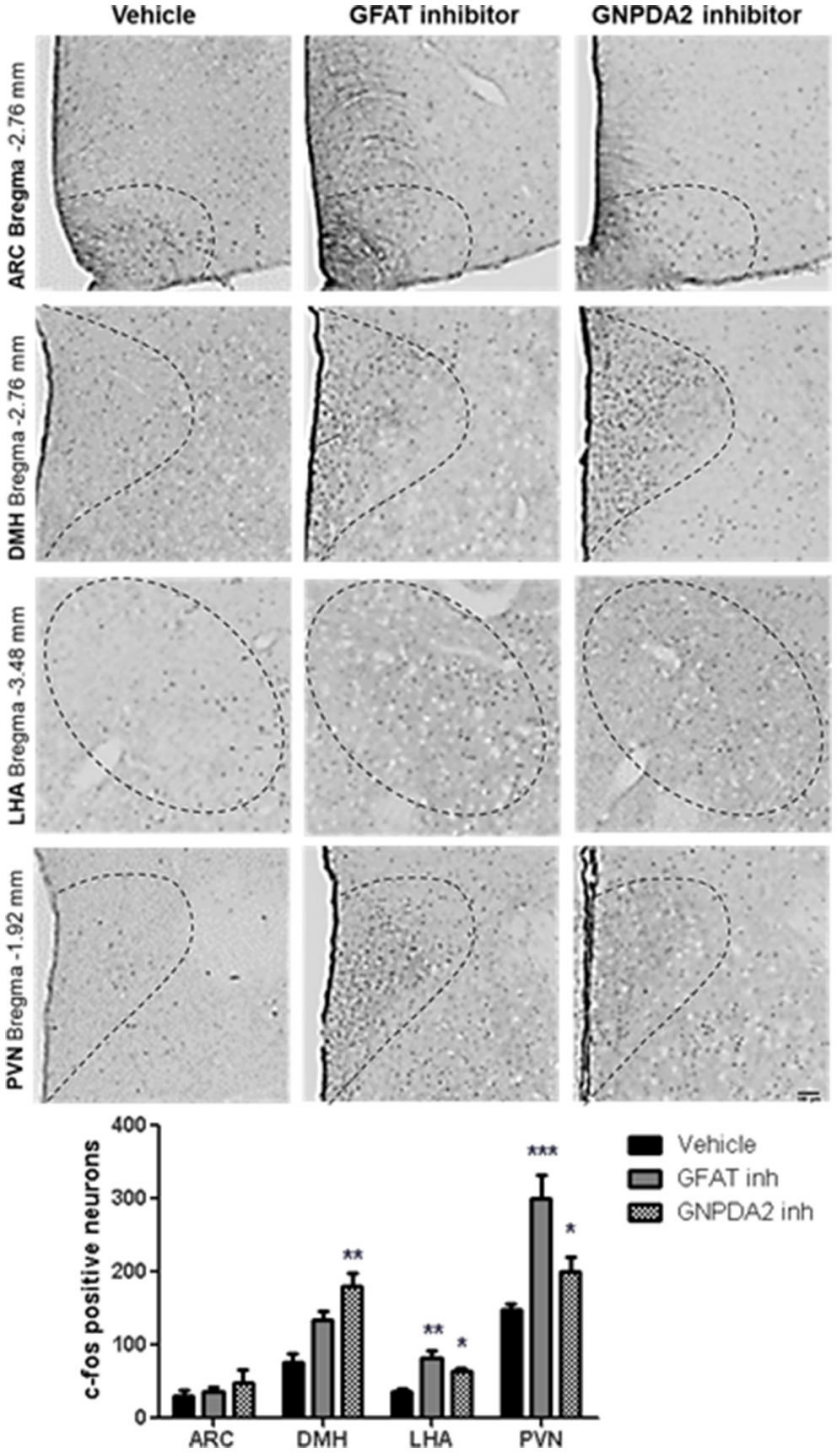
Neuronal activation by central infusion of GNPDA2 and GFAT inhibitors. Analysis of c-Fos positive neurons in ARC, PVN, DMH and LHA. Representative images of each hypothalamic nuclei. One-way ANOVA analysis, Tukey's *post-hoc* test. ^*^P < 0.05, ^**^P < 0.01, ^***^P < 0.001. *n* = 8 rats per group.

Once we identified that GNPDA2 is expressed in hypothalamus and its expression is controlled by nutrients, we decided to identify the anatomical location of neurons in the hypothalamus, activated by either GFAT and GNPDA2 inhibitors, that might be implicated in appetite control and glucose homeostasis. We used IHC to identify increased c-Fos as a marker of neuronal activity. After 1 h of injection of the compounds i3vt, we observed an induction of c-Fos in the PVN and LHA for both compounds compared to the controls. In the DMH, the induction was significant only for the GNPDA2 inhibitor and not for the GFAT inhibitor. Surprisingly, no difference was observed in the ARC (Figure 2).

### Dose-Dependent Effect of GNPDA2 and GFAT Inhibitors on Body Weight and Food Intake

**Figure 3:**
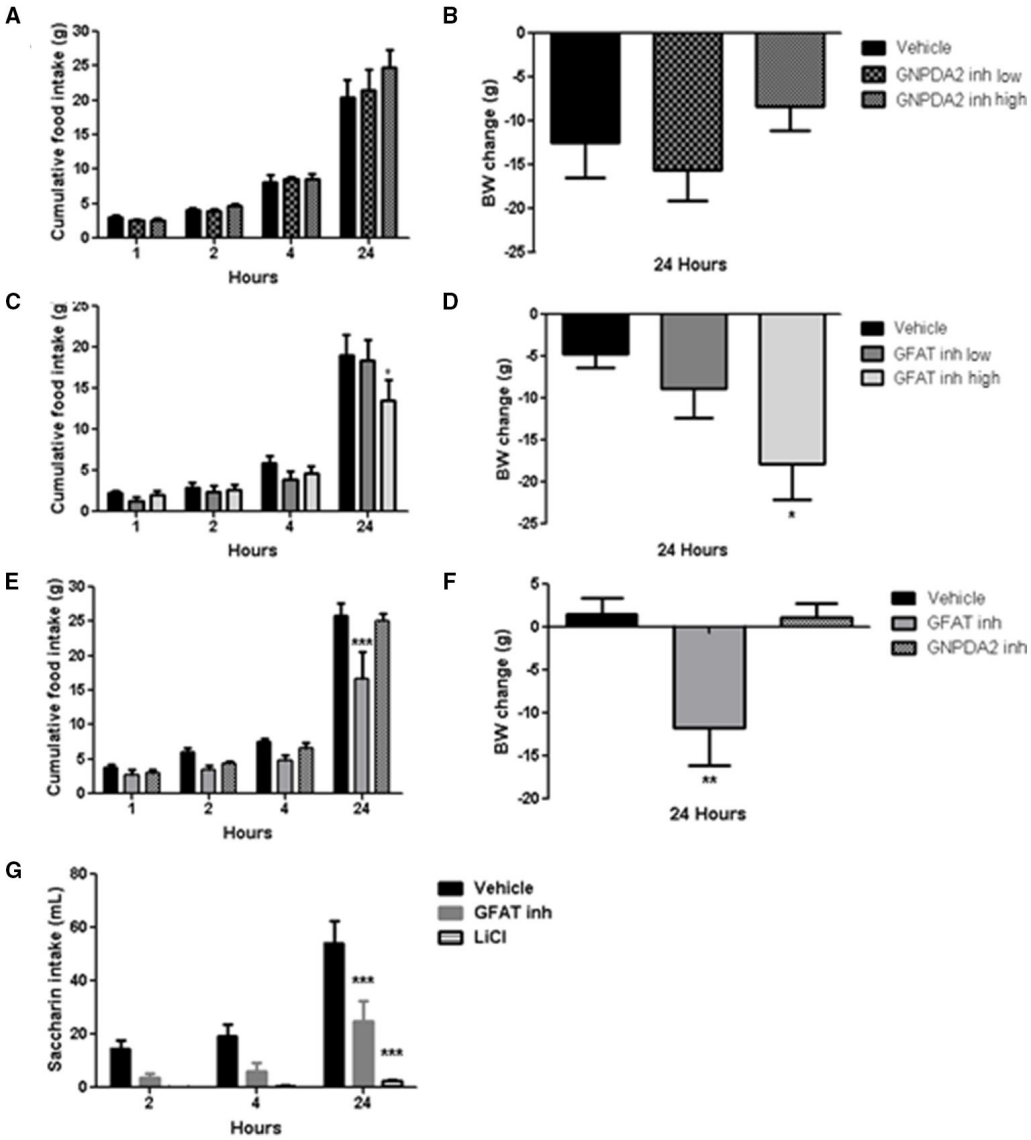
Impact of central infusion of GNPDA2 and GFAT inhibitors on food intake (FI) and body weight (BW). **(A, B)** Infusion of different concentrations of GNPDA2 inhibitor and its effect on FI and BW. **(C, D)** Infusion of different concentrations of GFAT inhibitor and its effect on FI and BW. **(E, F)** Infusion the same concentration of GNPDA2 and GFAT inhibitors (150 μg) and their effect on FI and BW. Repeated measures two- way ANOVA analysis for FI and one-way ANOVA for BW, ^*^P < 0.05, ^**^P < 0.01, ^***^P < 0.001. **(G)** Conditioned taste aversion test with GFAT inhibitor and LiCl. Repeated measures two- way ANOVA analysis, Bonferroni posttest, ^***^P < 0.001. *n* = 8 rats per group.

Acute dose of the inhibitors were injected 1 h prior to dark. Normally, animals will start eating during the dark period, so food intake at 1, 2, 4 h in the dark was measured to study the effect of the both enzymes over appetite control and the plausible long-lasting metabolic effect at 24 h. Two concentrations of the GNPDA2 inhibitor were infused i3vt 1 h before dark onset. There was no effect on either body weight or food intake (Figures 3A,B). In a similar fashion, two concentrations of the GFAT inhibitor (DON) were also infused, and both food intake and body weight were reduced at 24 h at the higher concentration (Figures 3C,D). In a follow-up experiment, in weight-matched rats, we administered the same concentration (150 μg) of both inhibitors i3vt. Consistent with the results in Figure 3C, food intake in the presence of the GFAT inhibitor was significantly reduced at 24 h; however, there was no effect of the GNPDA2 inhibitor (Figure 3E). As a consequence of the reduced food intake, the BW of the rats infused with the GFAT inhibitor was significantly reduced at 24 h (Figure 3F).

To determine whether the GFAT inhibitor provoked visceral illness, that might explain the reduced food intake, we performed a conditioned taste aversion test. Animals treated with LiCl (used as a positive control during the test) drank less saccharin relative to vehicle. Animals that had received the GFAT inhibitor also reduced their saccharin consumption relative to vehicle, implying that visceral illness had been provoked by the compound (Figure 3G). Thus, GNPDA2 does not regulate appetite and GFAT reduces food intake and body weight likely secondary to the visceral illness.

### Effects of Central GNPDA2 and GFAT Inhibition on Glucose Homeostasis

**Figure 4:**
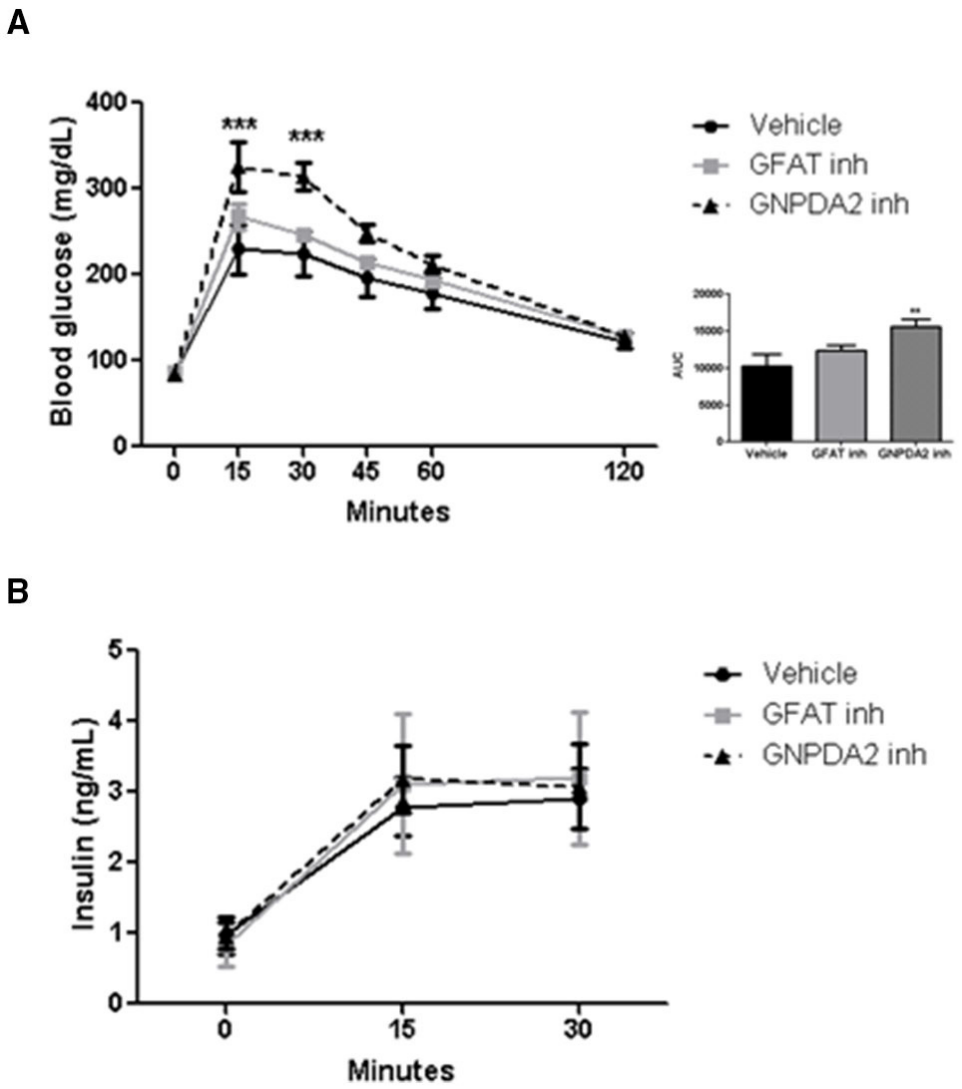
Impact of i3vt infusion of GNPDA2 and GFAT inhibitors on glucose homeostasis. **(A)** Central infusion of GNPDA2 and GFAT inhibitors (150 μg) 1 h prior to the intraperitoneal glucose tolerance test. **(B)** Insulin levels during the GTT. Repeated measures two-way ANOVA analysis, Bonferroni posttest ^***^P < 0.001, ^**^P < 0.01. *n* = 8 rats per group.

Increased HBP signaling plays a role in insulin resistance (19). To address the role of central GFAT or GNPDA2 in glucose homeostasis, we performed a glucose tolerance test (GTT) 1 h after infusion of the GFAT or GNPDA2 inhibitors i3vt. Animals treated with the GFAT inhibitor had comparable glucose tolerance to rats treated with saline. Interestingly, central inhibition of GNPDA2 provoked significant glucose intolerance (Figure 4A) without changes in plasma insulin levels (Figure 4B).

## Discussion

HBP signaling has been implicated in crucial aspects of cellular fuel sensing (2), but little is known about the function of GNPDA2, an important enzyme in this pathway. In *Escherichia coli*, GNPDA, homologous to human GNPDA2 and 1, has an important role on amino sugar utilization from environment and even from those derived from cell wall peptidoglycan recycling (20). However, in higher eukaryotes, its exact role remains undescribed.

An association of GNPDA2 with obesity and type-2 diabetes or both, has been reported in humans (8, 9). However, very little is known about how this enzyme might contribute to human susceptibility to obesity and type-2 diabetes. GNPDA2 is an important component of the HBP acting on the opposite direction for the rate limiting enzyme, GFAT. Given HBP's role as a crucial cellular fuel sensor pathway (2), one possibility is that GNPDA2 could be a neuronal nutrient sensor that links fuel availability to the regulation of energy and glucose homeostasis.

We first determined the expression of *GNPDA2* in different tissues and found that this gene is highly expressed in hypothalamus and adipose tissue of rats (Figure 1A) as reported in humans (9).

Until now, to our knowledge, only one paper has been published as the first approach to understand the function of GNPDA2 in metabolism. Wu et al. performed a transcriptome analysis in human adipose-derived mesenchymal stem cells after knockdown of *GNPDA2*, showing that these gene regulates lipid and glucose metabolism and could have a role in adipogenesis (11).

Therefore, we were interested in studying the effect of hypothalamic *GNPDA2* appetite control and glucose homeostasis as another approach to study its function. Given the increasing evidence that the hypothalamus integrates nutrient and hormonal signals to control mechanisms involved in energy balance and glucose regulation (21), we further analyzed the expression of this gene in different nuclei of the hypothalamus. We performed micropunches and found that it was expressed in several major nuclei of the medial basal hypothalamus that regulate food intake and glucose homeostasis (Figure 1B).

The diet-induced obesity animal model has been frequently used to underline the mechanisms implicated in regulating metabolism and energy balance and its influence in hypothalamic genes (22). For this reason, we analyzed the expression of *GNPDA2* and its opposite enzyme, *GFAT*, in the hypothalamus of rats fed chow and high-fat diet, as previously described (12). We observed that *GNPDA2* but not *GFAT* is down-regulated on HFD (Figure 1D). Another group reported no difference of *GNPDA2* expression under HFD in fasted mice. Moreover, it was more highly expressed in chow-fed compared to fasted mice (23). In our hands, the expression of these genes under fasted vs. fed conditions did not reveal any difference (Figure 1C). This discrepancy could be due to the different animal species (female mice vs. male rats), the duration of exposure to the diets (from weaning to 8 weeks were exposed to HFD vs. rats of 6 weeks old were exposed for 6 weeks to HFD), the type of diet (60% HFD plus 20% of sucrose vs. 60% HFD), or the nutritional state (fasted for 4 h vs. 48h). Thus, several important differences (species, sex and time of fasting) between these two studies might explain the variances of gene expression. Therefore, more studies have to be performed to better understand how the nutritional state influences the expression of *GNPDA2* and *GFAT* and their plausible implication in hypothalamic nutrient sensing.

It has been suggested that only 5% of the glucose goes into the HBP pathway (24). However, under enriched situations or an energy surplus, the HBP becomes enhanced leading to O-linked glycosylation of proteins involved in glucose responsiveness mechanisms and provoking desensitization of the insulin-responsive glucose transport system in adipocytes (24), and increasing triglyceride synthesis (25, 26) and glycogen biosynthesis (27), among others. Increased HBP in GFAT transgenic mice led to insulin resistance, but no additional effect was observed under HFD (28). In addition, HFD-fed animals had equal glucose disposal rates as transgenic GFAT mice; i.e., both groups are similarly insulin resistant (28). It is likely that the HBP flux is favored when animals are fed a HFD by leaving *GFAT* expression unaltered, but by down-regulating *GNPDA2* as observed in Figure 1C. As a consequence of overall enhanced HBP, an obese and insulin resistant phenotype emerges in animals fed a HFD.

Knowing that *GNPDA2* and *GFAT* are expressed in the hypothalamus and *GNPDA2* is regulated by nutrients, we then inhibited these enzymes to understand their role in appetite control and glucose homeostasis.

We observed that the GFAT inhibitor results in a reduction of food intake, leading to weight loss. However, whether this reflects a direct role of GFAT in the regulation of food intake is unclear, since we also observed evidence that the inhibitor also provoked visceral illness as evidenced by its ability to cause a conditioned taste aversion. The GFAT inhibitor did not have any effect on glucose homeostasis. The 6-Diazo-5-oxo-L-norleucine (DON), a glutamine analog, is broadly used to inhibit GFAT. However, it can inhibit other enzymes such as cytidine triphosphate synthase 1(s) and various glutaminases (29). The lack of specificity of this compound and other GFAT inhibitors (such as azaserine), contributes to the difficulty of assessing GFAT function *in vivo*. In addition, DON can be cytotoxic, which may have contributed to the visceral illness observed in the animals. In humans, it has already been reported that DON can cause nausea and vomiting (16). Therefore, more studies should be performed to clarify if the weight loss is due to visceral illness or to a direct impact of GFAT over nutrient sensing in the hypothalamus.

On the other hand, central inhibition of GNPDA2 did not have an effect on food intake and body weight. However, central inhibition of GNPDA2 did provoke significant glucose intolerance. A group studying the role of the glucokinase (GK) (the rate limiting enzyme in glycolysis and a glucose sensor) utilized glucosamine as a GK inhibitor (3, 4). GK phosphorylates glucose and facilitates conversion to F6P. F6P could then either continue toward glycolysis or go through the HBP. GFAT converts F6P and glutamine to GlcN-6-P (19). Alternatively, glucosamine can enter the HBP directly, bypassing GFAT where it is converted to GlcN-6-P. It was recently reported that centrally delivered glucosamine increases food intake (3, 4). In our results, we observed that central inhibition of GNPDA2 results in no significant change in food intake in the concentrations that we tested.

Interestingly, central GNPDA2 inhibition at a dose that modifies neither food intake nor body weight, the animals were glucose intolerant with no change of insulin secretion at 15 min (Figures 4A,B). When glucosamine was infused i3vt, there was an impairment of glucose handling due to a reduced insulin secretion (4). In that study, central administration of glucosamine impaired pancreatic first-phase insulin secretion (2–5 min after a glucose load) but at 15 min there was no difference (4). We did not observe impairment of insulin secretion at 15 min, potentially because we had already missed the first phase of insulin secretion. However, we nonetheless demonstrated that hypothalamic GNPDA2 modulates glucose homeostasis and it is possible that this regulation could be similar to the effects of glucosamine. The GNPDA2 inhibitor (13) could lead to increased flux through GlcN-6-P, being a plausible mechanism how this substance is leading to glucose intolerance and should be demonstrated in the future. These outcomes corroborate the results observed by Osundiji et al., where central manipulation of the HBP pathway leads to regulation of glucose homeostasis. In addition, our results demonstrate that inhibiting GNPDA2 directly in the CNS can have important effects on glucose homeostasis.

We did not investigate changes to extra-hypothalamic regions of the brain relevant to body-weight regulation, such as the limbic areas (i.e., amygdala, septal region and hippocampal formation), and area postrema/nucleus of the solitary tract. These areas are also uniquely sensitive to perturbations in glucose, either due to stress, memory consolidation, or projections directly from the visceral organs. However, we would predict these CNS regions would be sensitive to GNPDA2 inhibition directly and indirectly.

We have now demonstrated that pharmacological manipulation of the HBP pathway by GNPDA2 inhibition does not control appetite, but regulates glucose homeostasis. On the other hand, GFAT inhibition reduces food intake as a consequence of visceral illness. Interestingly, both enzyme inhibitors led to neuronal activation of hypothalamic nuclei that regulate appetite and glucose homeostasis. To our knowledge, this is the first paper that starts revealing the function of central GNPDA2 and GFAT in metabolism; however, more studies will have to be performed to understand its implication in obesity and type 2 diabetes.

Accumulating evidence points toward a crucial role for the hypothalamus to regulate glucose homeostasis in addition to its well described role in energy balance (1). Consistent with this, the HBP has been described as a nutrient sensing pathway. Then, the current work extends this by examining the regulation and impact of GFAT and GNPDA2 over appetite control and glucose homeostasis. In particular, the current results provide a potential explanation for the observation that GNPDA2 polymorphisms are associated with increased risk of type 2 diabetes. Importantly, the current work points toward a role for GNPDA2 in the CNS to regulate peripheral glucose levels.

## Data Availability Statement

The original contributions presented in the study are included in the article/supplementary materials, further inquiries can be directed to the corresponding author.

## Ethics Statement

The animal study was reviewed and approved by University of Cincinnati Institutional Animal Care and Use Committee (IACUC).

## Author Contributions

RG-A led the project, conceived, carried out the experiments, analyzed the data and drafted/revised the manuscript. BG, D-HK, and SY carried out experiments, helped with the data analysis and revised the manuscript. MC synthesized the GNPDA2 inhibitor. SW and RS contributed to the conception and interpretation of the data, drafted/revised the manuscript. All authors gave final approval of the manuscript to be published.

## Funding

This work was supported by NIH grants DK54080, DK056863, and DK 017844. GlcNol6P was synthesized using funds from PAPIIT-UNAM grant IN213312.

## Conflict of Interest

The authors declare that the research was conducted in the absence of any commercial or financial relationships that could be construed as a potential conflict of interest.

## Publisher's Note

All claims expressed in this article are solely those of the authors and do not necessarily represent those of their affiliated organizations, or those of the publisher, the editors and the reviewers. Any product that may be evaluated in this article, or claim that may be made by its manufacturer, is not guaranteed or endorsed by the publisher.
